# B4GALT5 high expression associated with poor prognosis of hepatocellular carcinoma

**DOI:** 10.1186/s12885-022-09442-2

**Published:** 2022-04-12

**Authors:** Yang Han, Zhe Li, Qi Wu, Hui Liu, Zhiqiang Sun, Yong Wu, Judong Luo

**Affiliations:** 1 Department of Radiotherapy, The Affiliated Changzhou No. 2 People’s Hospital of Nanjing Medical University, Changzhou, China; 2 Graduate School, Dalian Medical University, Dalian, China; 3 Department of Breast Surgery, Shuguang Hospital Affiliated to Shanghai University of Traditional Chinese Medicine, Shanghai, China; 4 Department of Histology and Embryology, Heze Medical College, Heze, China; 5 School of Computer Science and Technology, Nanjing Tech University, Nanjing, China; 6 Department of General Surgery, The Affiliated Changzhou No. 2 People’s Hospital of Nanjing Medical University, Changzhou, China

**Keywords:** Hepatocellular carcinoma, Prognostic biomarker, Immune infiltration level, Expression level, Survival analysis

## Abstract

**Background:**

B4GALT5 is postulated to be an important protein in sugar metabolism that catalyzes the synthesis of lactosylceramide (LacCer). However, its role in hepatocellular carcinoma (HCC) remains unknown.

**Method:**

We characterized the expression of B4GALT5 in HCC tissue compared to normal tissue, and explored its function of B4GALT5 in HCC by enrichment analysis based on its co-expressed gene set. Next, we checked whether B4GALT5 expression is correlated to immune infiltration level and clinical prognosis in hepatocellular carcinoma. Finally, we verified the expression of B4GALT5 using clinical samples evaluated by RT-PCR, and conducted in vitro experiments with B4GALT5-knockdown HCC cells to investigate the function of B4GALT5 in the HCC cell proliferation, migration and invasion.

**Results:**

We found B4GALT5 mRNA and protein expression levels were significantly high in HCC tissue compared to normal tissue. The enrichment analysis of the gene sets that co-expressed with B4GALT5 showed specificity in HCC-related pathways and functions. Also, the expression pattern of B4GALT5 was significantly related to the immune infiltration level, especially CD4+ T cell and macrophage cells. B4GALT5 higher mRNA expression was associated with poor overall survival (OS) in HCC patients. Furthermore, *In vitro* experiments showed that depletion of B4GALT5 significantly inhibited HCC cell proliferation, migration and invasion. This study revealed the function and its mediated pathways of B4GALT5 in HCC, indicating that B4GALT5 may serve as a prognostic biomarker of HCC.

## Background

Hepatocellular carcinoma (HCC) is the most common form of liver cancer and highly malignant with poor prognosis [1]. HCC has become a threat to global health, according to the global cancer report in 2018 [2]. More than 800 thousand new cases of HCC are reported worldwide each year, more than 87% HCC patients died and approximately half of them are Chinese [3]. It is a pressing demand to find novel prognostic biomarkers and therapeutic targets of HCC [4].Yet we know little about how it plays its role in HCC.

Aberrant glycosylation of receptors on cell surface often causes oncogenic transformation involved in the development and progression of tumor, including tumor cell proliferation, invasion, metastasis and angiogenesis [5]. Altered glycosylation of proteins is frequently attributed to abnormal expression of glycosyltransferases [6, 7]. As one of the seven beta-1,4-galactosyltransferase genes, B4GALT5 (beta-1,4-galactosyltransferase 5) catalyzes the synthesis of lactosylceramide (LacCer) via the transfer of galactose from UDP-galactose to glucosylceramide (GlcCer) [8, 9]. B4GALT5 is present on the cell surface and located in the Golgi complex, similar to other glycosyltransferases that function as adhesion molecules involved in matrix interactions, cell spreading and migration, and signal transduction cascades [10, 11]. Many studies have shown that glycosyltransferase is closely related to tumor occurrence and development [12, 13]. The relationship between B4GALT5 and several tumors, such as gynecological tumor and embryonic tumor, has been confirmed. However, the role of B4GALT5 in HCC has not been investigated to date.

In this study, we explored the expression and function of B4GALT5 in HCC, and examined the results using clinical samples and in vitro experiments. We found that B4GALT5 is significantly upregulated in HCC, and its overexpression is related to the poor prognosis of HCC patients. Also, the expression level of B4GALT5 is significantly related to the immune infiltration level in HCC, especially CD4+ T cell and macrophage cells. Our in vitro experiments showed that depletion of B4GALT5 significantly inhibited HCC cell proliferation, migration and invasion. In conclusion, B4GALT5 may be a potential biomarker for prognosis of patients with hepatocellular carcinoma.

## Results

### B4GALT5 is highly expressed in HCC and associated to poor prognosis

**Fig. 1:**
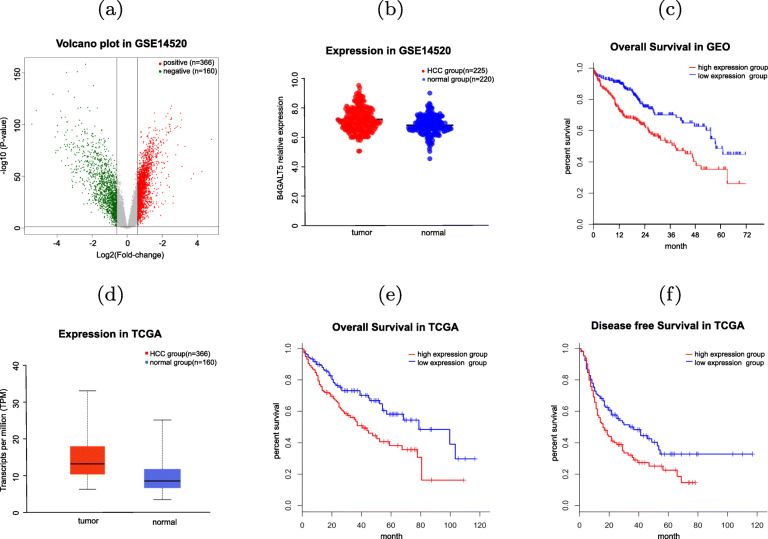
Differential expression and survival analysis of B4GALT5 in HCC. (**a**) Volcano plot of DEGs in HCC tissues and normal tissues in GSE14520 dataset. (**b**) B4GALT5 expression levels in HCC and normal tissues in GSE14520 dataset (*p*<0.05). (**c**) Kaplan-CMeier curves of overall survival of GSE14520 cohort grouped by B4GALT5 median expression level (*p*<0.05). (**d**) B4GALT5 expression levels in HCC and normal tissues in TCGA dataset (*p*<0.05). (**e**-**f**) Kaplan-CMeier curves of overall survival (OS) and disease-free survival (DFS) of TCGA cohort grouped by B4GALT5 median expression level (*p*<0.05)

We identified the differentially expressed genes (DEGs) between HCC tissues and normal tissues based on the GSE14520 dataset (T=225, N=220) and drew the volcano plot, as shown in Fig. 1 (a). The differential expression gene set included 2,273 upregulated genes and 1,458 downregulated genes (|*FC*|>1.5). Among the DEGs identified above, B4GALT5 was one of the genes with drastically increased expression in HCC tissues compared to normal ones. The survival analysis between high and low expression groups showed that high B4GALT5 expression indicate poor prognosis (Fig. 1 b-c).

We also used the LIHC dataset obtained from TCGA (T=366, N=160) to verify the association between B4GALT5 expression level and prognosis. Consistently, this also showed that the expression of B4GALT5 was higher in tumor samples than in normal samples (Fig. 1 d), suggesting its close relation with HCC. In addition, the patients with higher expression of B4GALT5 were found to have shorter overall survival (OS) and progression-free survival (PFS) than those in the normal group (Fig. 1 e-f). These results suggested that B4GALT5 high expression is unfavorable indicator of liver cancer patients.

### B4GALT5 conjugated with clinicopathological parameters improve prognosis accuracy

The mean expression level and statistic tests of B4GALT5 in different subgroup divided by other clinicopathological variables of HCC were listed in Table 1 on GSE14520 and TCGA, respectively. It can be found that the B4GALT5 only shows statistical difference in clinical stage subgroup (I-II vs. III-IV, *p*-value < 0.01) and gender subgroup (male *vs.* female, *p*-value < 0.05) on TCGA HCC cohort. On GSE14520 cohort, B4GALT5 only has different expression level in AFP subgroups (high *vs.* low, *p*-value < 0.05). The results showed that B4GALT5 is another independent biomarker of prognosis in HCC.

**Table 1:** Statistical correlation between B4GALT5 and clinicopathological parameters

Clinical	Group	No.	B4GALT5 median	*P*-value
TCGA				
age	>=60	175	4.453	0.442
	<60	160	4.536	
gender	F	108	4.689	0.0117
	M	227	4.399	
race	asian	155	4.511	0.0525
	black	14	4.397	
	white	164	3.673	
stage	I-II	247	4.381	0.0004
	III-IV	88	4.807	
GSE14520				
age	>=60	43	6.994	0.151
	<60	178	7.186	
gender	F	30	7.246	0.469
	M	191	7.133	
stage	I-II	170	7.094	0.072
	III-IV	49	7.322	
ALT	high	91	7.262	0.072
	low	130	7.069	
AFP	high	101	7.264	0.038
	low	117	7.044	
Multinodular	yes	45	7.325	0.093
	no	176	7.103	
Cirrhosis	yes	203	7.172	0.136
	no	18	6.883	

ALT: high(> 50U/L) and low (≤ 50U/L); AFP: high (> 300ng/ml) and low ≤ 300ng/ml)

**Fig. 2:**
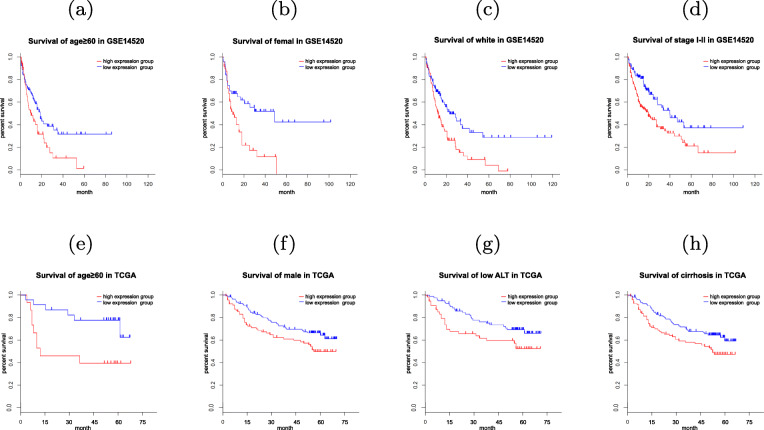
Survival analysis of B4GALT5 combined with clinicopathological parameters on TCGA cohort. (**a**-**d**) Kaplan-CMeier curves according to B4GALT5 signature with age ≥ 60, female, white, stage I-II patients in GSE14520 cohort(*p*<0.05). (**e**-**h**) Kaplan-CMeier curves of B4GALT5 signature with age ≥ 60, male, low ALT and cirrhosis patients in TCGA cohort(*p*<0.05)

Next, we checked whether B4GALT5 expression level conjugated with another clinicopathological variable can improve the prognosis accuracy. Survivals of GSE14520 cohort were shown in Fig. 2 (a-d), the overall survival status can be better speculated using B4GALT5 expression level for the age ≥ 60 patients. B4GALT5 expression level in female patients yield to more accurate prognosis. Especially,we observed statistically significant difference of survival status between the high and low B4GALT5 expression in white subgroup and early stage (I-II) subgroup. For TCGA cohort, B4GALT5 conjugated with age ≥ 60, male, low ALT and cirrhosis acquired significantly as shown in Fig. 2 (e-h). We supposed that B4GALT5 may be a promising biomarker of prognosis especially in certain subgroup of HCC patients.

### B4GALT5 co-expressed genes are enriched in HCC-related pathways and functions

**Fig. 3:**
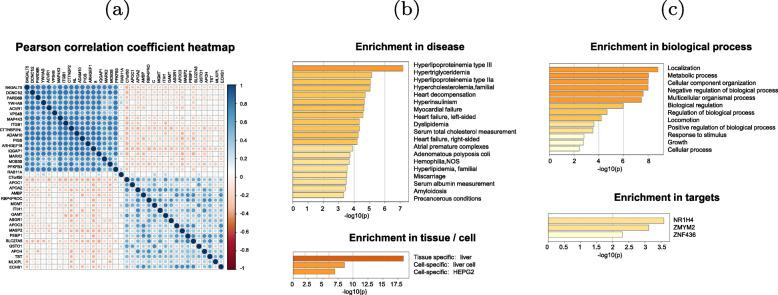
Enrichment analysis based on the set of B4GALT5 co-expressed genes in HCC. (**a**) The heatmap of the top 20 positively and negatively correlated genes of B4GALT5. (**b**-**c**) Bar plots of enriched terms in the DisGeNET10, PaGenBase, GO and TF-target knowledge repository of the set of B4GALT5 co-expressed genes

We identified the genes that were closely correlated with B4GALT5 based on the TCGA LIHC dataset. The genes with extremely low expression (*TPM*<0.5) in %50 samples were filtered out. Heatmap of the top 20 most positively and negatively correlated genes was shown in (Fig. 3 a). The set of co-expressed genes identified above were further used to explore the disease associations, gene patterns, GO functional enrichment and transcriptional factors by Metascape [14].

It can be found that B4GALT5 co-expressed genes are mainly enriched in the glucose-related diseases and lipid metabolism closely linked to HCC. Also, the set of co-expressed genes showed enrichment in liver tissue and HEPG2 cell line (Fig. 3 b). GO biological process were mainly concentrated in localization, metabolic process, growth and cellular process. Transcription factor targets included NR1H4, ZMYM2 and ZNF436, which might be metabolism-related and cancer-related factors (Fig. 3 c). These results further demonstrated that B4GALT5 is involved in the oncogenic pathways of HCC.

### B4GALT5 expression is correlated with immune infiltration level in HCC

**Fig. 4:**
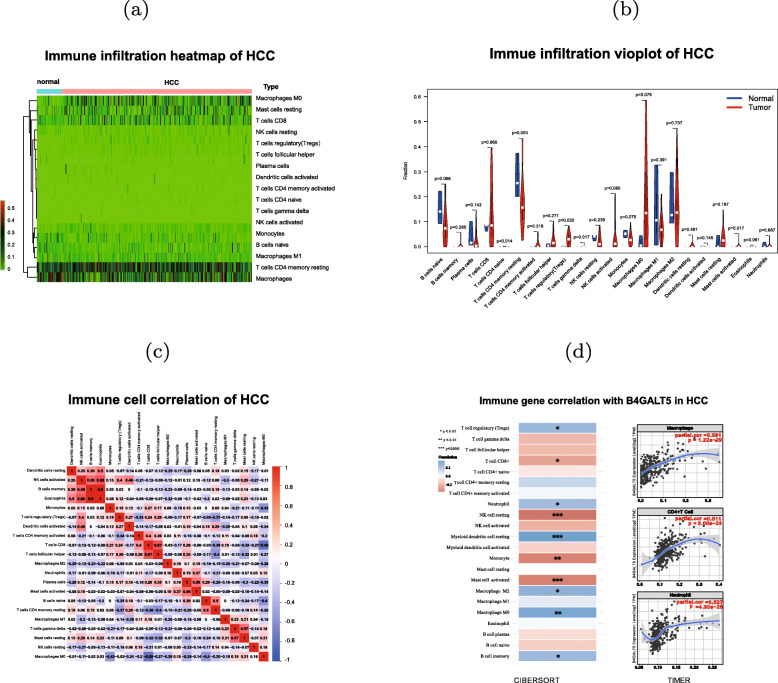
Immune cell counts in HCC tissues and association of B4GALT5 with immune infiltration level. (**a**) Heatmap of infiltrated immune cells in TCGA HCC samples. (**b**) Vioplot of expression of immune cells in HCC samples and normal samples. (**c**) Correlation heatmap of immune cells in HCC samples. (**d**) Correlation between B4GALT5 expression and immune infiltration levels evaluated by CIBERSORT and TIMER

We conducted immune infiltration analysis on TCGA LIHC dataset and explored the correlation between B4GALT5 expression and immune infiltration levels (n=371). As shown in Fig. 4 (a-b), the heatmap and vioplot showed that T-reg cells have high infiltration level in HCC tissues than in normal tissue (*p*-value < 0.05). The Correlation heatmap showed the dendritic resting cell, NK activated cells B memory cells showed significantly positive correlation (Fig. 4c).

Next, we investigated whether B4GALT5 expression is correlated with immune infiltration levels in HCC. Figure 4 (d) showed that B4GALT5 expression level had statistical correlation with the infiltration level of typical immune cells, particularly the NK resting cell, myeloid dendritic resting cell, mast activated cell. B4GALT5 expression level had significantly positive correlation with the infiltration level of macrophage cell, CD4+T cell and neutrophil. Table 2 results showed that B4GALT5 expression level was associated with immune cell infiltration. Especially, some types of immune cell infiltration in HCC had significantly higher correlation with B4GALT5 expression contrast to normal tissue, such as CD4 T, Monocyte and Th1 immune cells. These observations indicated that B4GALT5 played specific role in immune infiltration in HCC.

**Table 2:** Correlation between B4GALT5 expression and immune cell infiltration level on TCGA samples

Immune Cell	Subunit	Tumor group		Normal group	
		*c*	*p*-value	*c*	*p*-value
CD8+ T	CD8A	0.13	0.012	0.083	0.57
	CD8B	0.068	0.19	0.21	0.89
CD4+ T	CD3D	0.13	0.012	0.013	0.93
	CD3E	0.15	0.0051	0.03	0.84
	CD2	0.16	0.0019	0.029	0.84
B cell	CD19	0.066	0.21	-0.005	0.97
	CD79A	0.24	0.062	0.78	0.041
Monocyte	CD86	0.4	1.1E-15	0.31	0.029
	CD115	0.32	4.5E-10	0.11	0.45
M1	NOS2	0.009	0.86	-0.076	0.6
	IRF5	0.47	0	0.033	0.82
	PTGS2	0.27	2.2E-07	0.34	0.017
M2	CD163	0.15	0.0032	0.23	0.11
	VSIG4	0.21	6.6E-05	0.21	0.14
	MS4A4A	0.18	0.00039	0.16	0.27
Neutrophils	CD66b	-0.0048	0.93	-0.061	0.67
	CD11b	0.46	0	0.17	0.24
	CCR7	0.095	0.067	0.16	0.27
NK	KIR2DL1	0.051	0.33	-0.012	0.93
	KIR2DL3	0.13	0.014	-0.07	0.63
	KIR2DL4	0.23	6.6E-06	0.32	0.026
	KIR3DL1	0.05	0.34	0.2	0.17
	KIR3DL2	0.084	0.11	0.15	0.3
	KIR3DL3	0.11	0.04	0.0065	0.96
	KIR2DS4	0.0091	0.86	0.12	0.41
Th1	TBX21	0.12	0.022	0.093	0.52
	STAT4	0.31	2E-09	0.18	0.2
	STAT1	0.37	1.5E-13	0.025	0.87
	IFNG	0.13	0.015	0.16	0.25
	TNF	0.28	5.8E-08	0.19	5.8E-08
Th2	GATA3	0.25	7E-07	0.37	0.0073
	STAT6	0.33	1.3E-10	0.29	0.044
	STAT5A	0.36	0.044	0.23	0.044
	IL13	-0.039	0.45	0.27	0.059

### Immunohistochemistry and qRT-PCR validate higher B4GALT5 expression in HCC tissues

**Fig. 5:**
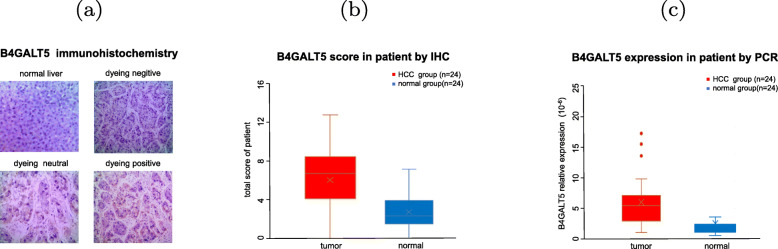
B4GALT5 protein abundance of B4GALT5 in HCC tissues. (**a**) B4GALT5 protein abundance in normal, negative, neutral and positive expression in HCC tissue by IHC. (**b**) B4GALT5 protein level measured by IHC in HCC tissues and adjacent normal tissues(*p*<0.05). (**c**) B4GALT5 protein level measured by PCR in HCC tissues and adjacent normal tissues (*p*<0.05)

The protein level of B4GALT5 in HCC tissue and matched adjacent tissue samples were analyzed using immunohistochemistry experiments. Figure 5 (a) showed different results of immunohistochemical staining. According to the total scores analysis, HCC tissues exhibited higher B4GALT5 protein expression level than paracarcinoma tissues (Fig. 5 b). Similarly, B4GALT5 mRNA expression levels were analyzed in the HCC tissues and paracarcinoma tissues by PCR, the results showed that the mRNA expression of B4GALT5 HCC tissues was higher than that in paracarcinoma tissues (Fig. 5 c).

### Knockdown of B4GALT5 significantly reduces proliferation, migration and invasion of HCC cells

**Fig. 6:**
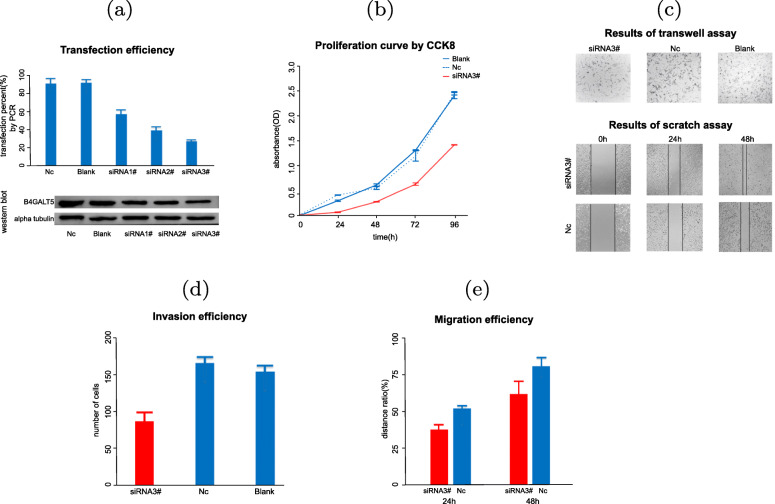
siRNA-induced Knockdown of B4GALT5 affect the capacity of proliferation, migration and invasion of HCC cells by in vitro experiments. (**a**) B4GALT5 transfection efficiency of liver cells evaluated by PCR and western blot. (**b**) Proliferation curve of CCK8 assay between B4GALT5 knockdown and control group (*p*<0.05). (**c**) Snapshot of transwell assay and scratch assay among B4GALT5 knockdown, control and blank group. (**d**-**e**) Invasion and migration efficiency of B4GALT5 knockdown cells by transwell assay (*p*<0.05)

To explore the function of B4GALT5 in HCC cells, we transfected siRNA into HCC cells and employed RT-qPCR and western blot to verify that the mRNA level of B4GALT5 was significantly decreased following si-B4GALT5 #3 transfection compared with si-NC transfection (Fig. 6 a). We then used these transfected cells for cell functional experiments. Cells transfected with #3 si-B4GALT5 exhibit significantly decreased proliferation compared with those in the control group (si-NC) and blank group (Fig. 6 b). Transwell and scratch assays showed that the number of invaded cells and the migration capacity of transfected cells are less than those cells in the control group, indicating that the invasive and migratory capacities of the cells with knockdown B4GALT5 were reduced (Fig. 6 c-e).

## Discussion

The malignant degree of HCC is high while surgi- cal treatment is only applicable for early-stage patients. Although various treatments, such as transcatheter arterial chemoembolization/(TACE), radiotherapy and locoregional therapy [15], have been developed, HCC still has a high mortality rate. The advanced stage and frequent recurrence after surgical resection are the main reasons of high mortality rate of HCC. Another reason lie in that the majority of the patients were limited to surgery or chemotherapy [16]. A few factors that are responsible for the initiation of HCC has been investigated, the underlying mechanisms for the conversion of healthy hepatic cells to neoplastic cells are still undefined. Therefore, early diagnosis and prognostic biomarkers are the focus of liver cancer research persistently [17].

Studies have shown that abnormal changes of glycosyltransferase are closely related to the occurrence and development of tumors [18]. In tumor cells the function of glycosyltransferase is aberrant because of genetic alterations and/or epigenetic modifications, which results in abnormal extracellular matrix and cell-cell interactions [19]. As aberrant glycans is involved in promoting tumor progression and metastasis [20], these enzymes are considered as therapeutic targets. In fact, genetic alterations in some of these glycotransferases have been gradually confirmed to be closely associated with liver cancer. For example, FUT8 activates PI3K-Akt-NF- *κ*B signaling pathway and promotes the proliferation of HCC cells, it also stimulates the expression of drug-resistant proteins. Inhibition of FUT8 can weaken hepatocyte epidermal growth factor (EGF) and hepatocyte growth factor (HGF), and thus reduce the incidence of liver cancer [21, 22]. miR-23a may affect the formation of N-glycochain branches on the cell surface through glucose transferase MGAT3, thereby increasing the metastasis potential of liver cancer, which provides a new idea for the mechanism of action of MGAT3 in tumor metastasis [23, 24]. B4GalT5 participates in the synthesis of both N-linked oligosaccharides and various glycolipids, it involves in extraembryonic development and tumorigenesis in many kinds of tumors, such as astrocytoma, glioma, breast cancer and cervical cancer [9, 25–27]. In fact, B4GalT5 has been reported to be one of the target genes connected to adverse clinical outcomes in patients with HCC [28].

This study sets about to clarify the role of B4GALT5 expression and its prognostic significance in HCC, by combining large-scale cohort analysis and in vitro experiments. On the one hand, glycosylation is closely related to glucose and lipid metabolism and insulin resistance, which promotes the occurrence and development of liver cancer [29]. Both our results and previous studies indicate this point. For instance, the metabolic status in mice after B4GALT5 gene knockout is improved [8, 30]. Moreover, glycosylation is related to tumor immune microenvironment which leads to poor prognosis [31]. Our results show that the degree of immune infiltration level in HCC is significantly correlated with B4GALT5 expression, especially in macrophages and neutrophils. Our finding is actually consistent to the conclusions of previous reports. For instance, the precursor of B4GALT5 *β*-GlcCer and its product LacCer are shown to be associated with tumor immune microenvironment. *β*-GlcCer was identified as an endogenous ligand for Mincle and possess immunostimulatory activity [32], while LacCer and kinase Lyn that form microdomains rich in spingolipids on the plasma membrane of neutrophils play an important role in innate immune function of neutrophils [33, 34]. In fact, many studies have suggested that B4GALT5 may be an inflammatory factor and a regulator of M1 infiltration, which increases inflammation and insulin resistance [35, 36]. As the regulation and functional mechanism of B4GALT5 still remains unclear, our future work will focus on the inflammatory reaction and immune regulation to reveal the role of B4GALT5 in HCC.

## Conclusion

In this study, we demonstrated for the first time that B4GALT5 is over-expressed in HCC and positively associates with poor survival of HCC patients. We also found that B4GALT5 combined with another clinicopathological parameter can improve prognosis accuracy. The gene set enrichment analysis showed that B4GALT5 co-expressed genes were enriched in HCC-related pathways and functions. We also conducted immune infiltration level analysis and confirmed the association of B4GALT5 with tumor immune microenvironment in HCC. Next, we used clinical samples and cell experiments to verify the function of B4GALT5 in HCC cells. Our findings was actually consistent with the conclusions of previous studies that high B4GALT5 expression is a biomarker of poor prognosis of HCC patients. As a result, we suggested that inhibition of B4GALT5 may be a promising therapeutic target to treat HCC.

## Methods

### Expression and survival analysis of B4GALT5 in HCC

The gene expression dataset of human hepatocellular carcinoma was obtained from GEO GSE14520 [37]. This dataset included 239 HCC tumor samples and 249 normal samples. The R package *limma* [38] was used to run differential expression analysis. The volcano plot were produced by *limma*. The survival analysis for differentially expressed gene set were analyzed using Graphpad Prism (version 8.2.1 Windows version, GraphPad Software, San Diego). Another dataset obtained from TCGA LIHC project [39] contained 371 tumor samples and 50 normal samples was used to verify gene expression and prognosis.

### Survival analysis based on B4GALT5 coupled with clinicopathological parameters

The clinical features of HCC patients were downloaded from GEO and TCGA. Each cohort was grouped according to different features, and then the mean expression levels of B4GALT5 in each subgroups were computed. The *p*-value was obtained by group *t*-test. A three-line table was used to show the relationships between clinical feature and B4GALT5 expression. Next, for each subgroup of patients divided by a clinical feature, survival analysis was run on the subgroups stratified by mean B4GALT5 expression level. The curves of survival analysis with statistical significance were presented.

### Enrichment analysis of B4GALT5 co-expressed genes

The co-expressed genes of B4GALT5 were selected based on Spearman correlation coefficient, using the criteria of *p*-value less than 0.01. The set of co-expressed genes were filtered and clustered based on their membership similarities. Top 20 positively and 20 negatively co-expressed genes were used for further enrichment analysis. Metascape [14] was used for functional analysis on the set of co-expressed genes. The results included gene set enrichment analysis in disease association in DisGeNET database [40], transcription factor targets, and tissue/cell type association in PaGenBase database [41].

### Immune infiltration level analysis

The immune-related gene expressions in HCC were screened based on gene expression matrix. The heatmap, vioplot and correlation plot of immune cells were drawn by R package. To further identify the relationship between B4GALT5 and immune infiltration level, the Spearman correlation of B4GALT5 expression with typical immune cells, including B cells, CD4+ T cells, CD8+ T cells, neutrophils and macrophages, was calculated.

The R package *immunedeconv* [42] was used to evaluate the scores of immune cells with different B4GALT5 expression. CIBERSORT [43] and TIMER [44] algorithms were both used to estimate immune cell count in HCC tissues. TIMER considered tissue specificity in the estimation of immune cell counts, while CIBERSORT can assess T cell characteristics in more detail (* *p*<0.05, ** *p*<0.01, *** *p*<0.001). The correlation between B4GALT5 expression and immune cells were presented in tabular form. *p*-value less than 0.5 is considered as statistical significance between the infiltration level of immune cell and B4GALT5.

### Immunohistochemistry experiments

A total of 24 hepatocellular carcinoma tissue samples and matched adjacent normal tissue samples were acquired from the Affiliated Changzhou No. 2 People’s Hospital of Nanjing Medical University (Jiangsu, China). All tissues were obtained by surgery. The histology of the 24 hepatocellular carcinoma tissue specimens was confirmed by the senior pathologist of the Department of Pathology at the hospital. First, tissue sections were treated for antigen retrieval using EDTA buffer, and then the tissues were incubated with a primary anti-B4GALT5 antibody (1:200, Biorbyt) at 4^∘^C overnight. Next, the sections were incubated with a secondary antibody and then visualized using diaminobenzidine (DAB) and hematoxylin counterstaining after washing with PBS. The immunohistochemical results were evaluated by pathologists and scored as follows: 0, negative; 1, +; 2, ++; and 3, +++. The positive staining rate was defined according to the proportion of positively stained cancer cells: 0, negative; 1, 1-20%; 2, 21-40%; 3, 41-60%; 4, 61-80%; and 5, 81-100%. The staining intensity score plus the staining positive rate score is counted as the total score.

### Quantitative real-time PCR

Total RNA was isolated from cells using an RNA extraction kit (centrifugal column assay) according to the manufacturer’s instructions. Quantitative RT-PCR (q RT-PCR) was performed on an ABI ViiA7 Series PCR instrument (Applied Biosystem, ThermoFisher, USA) using FastStart Universal SYBR Green Master Mix (ROX) (Roche, Sigma-Aldrich). The primer sequences were as follows: B4GALT5, forward 5’-TCCTCGCTGCTGTACTTCG-3’ and reverse 5’-AATGCCTTGGGCTTGCATCA-3’; 18S (serving as the internal reference), forward 5’-TCCTCGCTGCTGTACTTCG-3’ and reverse 5’-TTACAGGGCCTCGAAAGAGTCC-3’. Reactions were carried out at 95^∘^C for 30s, followed by 40 cycles at 95^∘^C for 5s and 60^∘^C for 30s. The fluorescence data were collected in the 60^∘^C extension phase. Each sample was measured in 3 technical replicates.

### Western blot

Western blot was used to verify the transfection efficiency.The total protein of cells was extracted using radioimmunoprecipitation assay (RIPA) lysis buffer. The intact protein was separated by polyacrylamide gel electrophoresis (PAGE) and transferred onto a nitrocellulose membrane using a wet transfer method. The membrane was blocked with 5% BSA for 1h and then probed at 4^∘^C overnight with primary antibodies specific for B4GALT5 (1:1000, Bioworld, 45 KD) or Alpha tubulin (1:2000, 55 KD); Alpha tubulin served as the internal reference. After washing with TBST, the membrane was incubated with horseradish peroxidase (HRP)-labeled goat anti-rabbit IgG (1:10,000), washed with TBST, developed with an enhanced chemiluminescence (ECL) solution, and imaged for analysis,and the greater the amount of target protein, the less obvious the knockdown efficiency.

### Cell culture and transfection

SK-Hep-1 human hepatocellular carcinoma cells were cultured in DMEM (Gibco, USA) supplemented with 10% FBS (Gibco, USA) in 5% CO2 at 37^∘^C. B4GALT5 silencing was performed using three custom-made siRNAs targeting the B4GALT5 mRNA region (siB4GALT5 #1 sense: GCUGCUGUACUUCGUCUAUTT, siB4GALT5 #1 antisense: AUAGACGAAGUACAGCAGCTT; siB4GALT5 #2 sense: GGAAGCCUUCUGAUUGCAUTT, siB4GALT5 #2 antisense: AUGCAAUCAGAAGGCUUCCTT; and siB4GALT5 #3 sense: CCAGUUUCUUGGAAGGUAUTT, siB4GALT5 #3 antisense: AUACCUUCCAAGAAACUGGTT) and Nc control siRNA (GenePharma, China). Cells in the exponential growth phase were plated in a six-well plate(5 × 10^5^ cells/well) and transfected with siRNA after 24h by Lipofectamine 3000 Transfection Reagent following the manufacturer’s instructions. For validation, we extracted total RNA, on which the described RT-PCR was performed, and Western blotting of the total proteins from transfected cells was performed.

### Cell proliferation, invasion and migration assays

Transfected cells and control cells in the logarithmic growth phase were harvested. They were seeded in 96-well plates (1000 cells/well) with five replicates per sample and incubated at 37^∘^C, and were quantified every 24h using Cell Counting Kit-8 (Dojindo, Kumamoto, Japan). The absorbance was measured at 450 nm for proliferation analysis. A transwell assay was used to evaluate cell invasion. A total of 5 × 10^4^ cells were seeded in the upper chamber with serum-free culture medium (200 *μ*l) after the upper chamber was coated with Matrigel (BD Biosciences, San Jose, CA), and the lower chamber was filled with 20% FBS medium. After culturing for 24h, the cells were fixed with 4% paraformaldehyde and stained with crystal violet for 15 min.The migrated cells were counted in random fields. A scratch assay was used to analyze the migratory ability of cells. After 24h of transfection, cells were seeded in a 6-well plate at 1 × 10^6^ cells/well. When the cells were approximately 95% confluent, they were vertically and linearly scratched with a 20 *μ*l micropipette. After washing to remove the nonadherent cells, the cells were cultured with serum-free culture medium and imaged at 0 h, 24 h and 48 h. The cell migration distance was measured using Image-Pro Plus Analysis software.

### Statistical analysis

Survival curves were plotted by the Kaplan-Meier (KM) method,univariate and multivariate analyses were performed using the Cox proportional hazards model. We used the Chi-square/Fisher’s exact test for categorical variables and the two-sample t-test/Wilcoxon rank-sum test for continuous variables to observe the statistical significance. Unless otherwise specified, the 2-tailed *p*-value less than 0.05 was considered statistically significant.

## Data Availability

The datasets generated and analysed during the current study are available in the GitHub repository at https://github.com/hliu2016/B4GALT5.
